# Qualitative, energy and environmental aspects of microwave drying of pre-treated apple slices

**DOI:** 10.1038/s41598-023-43358-6

**Published:** 2023-09-26

**Authors:** Ebrahim Taghinezhad, Mohammad Kaveh, Antoni Szumny, Adam Figiel, José Blasco

**Affiliations:** 1 Department of Agricultural Technology Engineering, Moghan College of Agriculture and Natural Resources, University of Mohaghegh Ardabili, 5619911367 Ardabil, Iran; 2 Department of Chemistry, Wroclaw University of Environmental and Life Science, CK Norwida 25, 50-375 Wrocław, Poland; 3 Department of Petroleum Engineering, College of Engineering, Knowledge University, 44001 Erbil, Iraq; 4 Institute of Agricultural Engineering, Wroclaw University of Environmental and Life Sciences, Chełmońskiego 37a, 51-630 Wrocław, Poland; 5 Centro de Agroingeniería, Instituto Valenciano de Investigaciones Agrarias (IVIA), CV-315, Km 10.7, Moncada, 46113 Valencia, Spain

**Keywords:** Devices for energy harvesting, Climate change, Statistics

## Abstract

In the present research, response parameters such as specific energy consumption (*SEC*), thermal efficiency (*TE*), energy efficiency (*EF*), drying time (*DT*), greenhouse gas (GHG) emission (such as CO_2_ and NO_x_), and quality features (color variation and shrinkage) were modeled by response surface methodology (*RSM*) for apple slices dried in a microwave dryer under ultrasonication (30 ℃—10 min) and blanching (80 °C—2 min) pretreatments. Also, *RSM* was applied to optimize two independent parameters including microwave power and sample thickness in the levels 100, 200, and 300 W and 2, 4, and 6 mm, respectively. The results indicated the significant influence (*P* < 0.01) of the independent parameters on the response parameters. The vales of *SEC*, *DT*, GHG emission, shrinkage, and color difference were linearly decreased with the declining sample thickness and increasing microwave power, while the energy and thermal efficiencies were increased by a quadratic equation. The use of ultrasonication and blanching pretreatments decreased the *SEC*, GHG emissions, and *DT*; while improving the quality of the samples as compared to the non-treated slices. The optimization results showed the optimal drying times (31.55, 82.19, and 50.55 min), *SEC* (3.42, 10.07, and 4.37 MJ/kg), CO_2_ with natural gas (1539.75, 1518.75, and 4585 g), CO_2_ with gas oil (3662.53, 2099.25, 2721.25 g), NO_x_ with natural gas (10.094, 9.956, and 12.906 g), and NO_x_ with gas oil (12.934, 12.758, and 16.538 g) at a microwave power of 300 W and sample thickness of 2 mm with desirability of 0.921, 0.935, and 0.916 for control samples, ultrasonicated, and blanched, respectively.

## Introduction

The apple has a wide application in the food industry and has consistently been in the focus of the consumers. The annual apple production amounted to 86,442,716 tons from 4,622,366 ha of the orchards in 2020^1^. Therefore, regarding the high annual production amounts of this product, storage of apple is of crucial significance. The apple can be eaten fresh or as dried chips. The storage life of this product can be significantly prolonged with preserved quality by decreasing its moisture content (*MC*) through the drying processes^2–4^. Despite the remarkable advantages of drying in the processing and storage of food and agricultural products, the use of inappropriate methods under undesirable conditions can decrease the quality value of the final product along with a high energy consumption and increased production costs^5,6^. Quality characteristics such as color and shrinkage of food and agricultural products are among the most popular and influential factors that determine consumers' acceptance of them^7^. The protection of product quality with minimum specific energy consumption (*SEC*) is the most important challenge in the drying industries^8^. Therefore, the *SEC* of the drying process and the final quality of the samples have to be evaluated to achieve optimal drying conditions^9^ To solve these problems, a microwave dryer was applied to dry apple slices. Currently, there is an emerging trend of using new drying methods, such as microwave drying, which significantly increases the drying rate and product quality, and reduces energy consumption and greenhouse gas (GHG) emissions^10^. In contrast with other conventional thermal methods, heat is spread throughout the product in the microwave drying method because of the penetration of microwaves into the food^11^. Microwave drying results in desirable quality of the dried products such as larger water resorption, lower shrinkage, and higher porosity. Furthermore, this drying method can save *SEC* because of the shortening of the process time^12^. Other drying processes are accompanied by high *SEC*, accounting for 12–20% of global energy consumption^13^. Several reports describe emerging dryers (including microwave dryers) on energy indices such as energy efficiency (EF), thermal efficiency (TE), SEC, and drying efficiency (DE) for various crops such as garlic in a microwave vacuum^14^, gala apple in a microwave dryer with blanching, electric field and freezing pretreatment^15^, and *Rosmarinus officinalis* L in a convective and hybrid vacuum microwave^16^.

One of the most common non-thermal techniques to maintain the quality properties of food used as a pretreatment or in combination with different drying methods is ultrasound (US)^17^. In recent years, ultrasound pretreatment has become one of the most common mechanical pretreatments among various pretreatments (blanching, cold plasma, radio frequency, ethanol, Citric acid, potassium metabisulphite, ohmic heating) and has shown acceptable results in terms of increasing the drying properties of fruits and vegetables and reducing energy consumption^18^. In food processing, ultrasound causes changes in textural, rheological, physical, chemical and functional properties^19^. Several studies have also dried various food products using ultrasound pretreatment for *Inula viscosa* (L.)^17^, pumpkin seeds^20^, longan^21^, apple^22^, celery^23^, carrot^24^, and kiwifruit^25^. The results showed that pretreatments with different drying methods can be applied to decrease and increase the *DT* and the efficiency, respectively while preserving the final sample quality.

Today, due to the increase in population, the reduction of arable land and the improvement of the standard of living, the amount of energy consumption in the agricultural sector has increased^26^. In order to provide food for the growing population, intensive use of agricultural machines, electrical energy, and natural resources is needed. But because fossil resources are limited, and need to be preserved for future human generations, it is necessary to use them correctly and with high efficiency^27^. On the other hand, the increasingly intensive use of energy resources causes environmental problems^28^. During the last decades, the concentration of greenhouse gases has increased rapidly in the earth's atmosphere. In dry fruit production, drying has a large share of greenhouse gas emissions, hence strategies should be adopted to optimize energy consumption in the drying sector. Karimi et al.^29^ showed that using temperature from 40 to 60 °C and increasing Heat Carrier Particle (HCP) to seeds from 0 to 0.5 for drying Canola seeds in a fluidized bed dryer reduces GHG emission. Kaveh et al.^30^ compared different drying methods (hot air, hot air-infrared, hot air-microwave, hot air-solar, continuous conveyor) to reduce the GHG emissions of *Pistacia Atlantica* drying and reported that the combined microwave-hot air method produces the lowest amount of greenhouse gas. In another study, Miraei Ashtiani et al.^31^ stated that the use of cold plasma pretreatment before drying goldenberry in an ultrasound-assisted convective dryer leads to the reduction of GHG emissions. Motevali et al.^32^ suggested the microwave-hot air method for drying Aloe vera in different ways to reduce the amount of GHG emissions. Seyfi et al.^33^ from the comparison between three types of hot air, refractance window, and solar assisted refractance window based on the photovoltaic-thermal system dryers in drying Aloe vera gel, came to the conclusion that increasing the temperature and decreasing the thickness causes the reduction of GHG emissions.

Optimization means improving the performance of a system, process or production method in order to achieve maximum benefit from them*. RSM* includes a series of statistical and mathematical techniques to describe the relationships between the independent and response variables by mathematical equations and optimization of these responses^34^. The purpose of applying this method is to find the best set of operating levels to achieve some specific and desired characteristics and also to optimize various processes. Kaveh et al.^35^ optimized the qualitative (color and shrinkage) and quantitative (drying time, effective moisture diffusivity, and specific energy consumption) properties of the kiwifruit with sample thicknesses, microwave powers, and ultrasound pretreatment times with the response surface method. They showed that thickness of 4 mm, power of 257.2 W, and ultrasound time of 30 min is the optimum point for drying kiwi. In another study, Liu et al.^36^ optimized the different properties of purple cabbage drying in combined microwave/hot air dryers using the response surface method (Box–Behnken design). Their results showed that microwave density of 2.35 W/g, moisture content of conversion point at 4.0 g/g, and hot air temperature at 55 °C were chosen as the optimum points.

Literature review has shown that no research has reported the application of *RSM* to optimize the drying variables and model the thermal and quality properties of dried apple slices in a microwave dryer under different pretreatments. Therefore, the scope of this research is to optimize and model the dependent (color variation, shrinkage, *EF*, *SEC*, *GHG* emissions, *TE*, *DT*, and dryer efficiency) and independent (microwave power and sample thickness) variables of drying apple slices by a microwave dryer using ultrasonication and blanching pretreatments with the help of *RSM*.

## Materials and methods

The Golden delicious variety of apples was harvested from the identified orchards in Moghan college, Ardabil province, Iran in November 2021. The collection of apple samples and the performance of experimental research on such fruits complied with all the relevant Moghan college guidelines and legislation. Also, the use of fruits in the present study complies with national guidelines of Iran National Standards Organization^37^. The apples were first sorted uniformly according to the ripeness degree. Then the fruits were kept in the refrigerator at 4 to 6 °C to prevent rotting. Samples were placed at ambient temperature for 2 h before the tests^22^. The apples were washed and peeled then cut into slices of 2, 4, and 6 mm thickness with a knife. The initial *MC* of the fresh apple was obtained by an oven (Memmert Company, UFB500, Schwabach, Germany) at 103 $^\circ{\rm C}$ for 24 h in triplicate. Based on the results, it was 83.2 ± 1% (w.b.).

### Ultrasonication and blanching pretreatments

An ultrasonic bath (Parsonic, model 7500S, Iran) at 28 kHz frequency was applied for ultrasonication. The sliced fruits were subjected to ultrasound waves at air temperature of 30 °C for 10 min in a distilled water^38^. The ratio of the distilled water to the sample was 4:1, such that all the samples uniformly received the ultrasound waves^39^. For blanching, the apples slices were subjected to the vapor of distilled water at the constant temperature of 80 °C for 2 min^40^. After pretreatment and removing the surface water with a towel tissue, the samples were placed in a glass cylinder-shaped container (in a thin layer) and dried with a microwave dryer until they reached a constant weight.

### Microwave dryer

**Figure 1:**
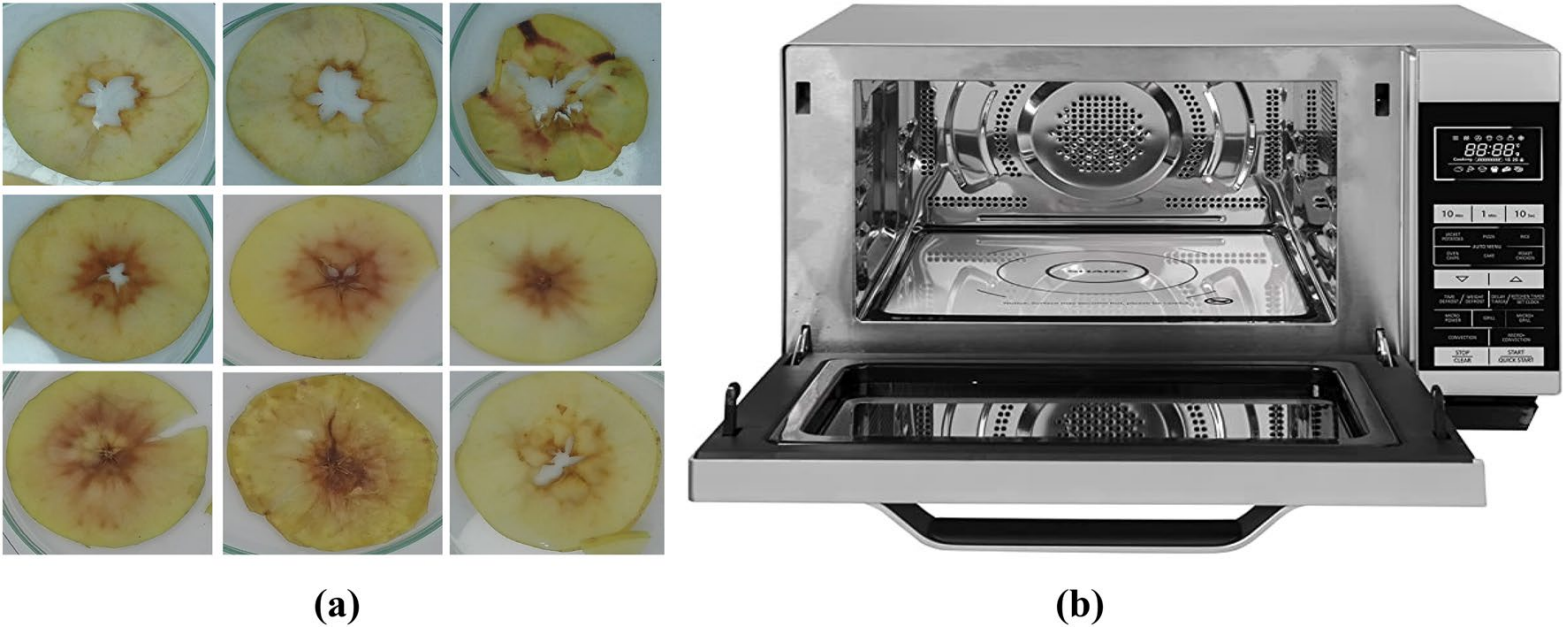
(**a**) Dried samples which were randomly selected from different treatments. (**b**) Microwave dryer.

According to Fig. 1, a commercial domestic microwave (Sharp company, model R-861SLM) was applied to dry the apple slices with three thicknesses of 2, 4, and 6 mm at three microwave power of 100, 200, and 300 W. Samples of 80 g of apple slices were prepared for each test. A layer of the pretreated and control (untreated) samples was placed in the microwave container. The tray with the slices was on a digital balance (from the AND company, model GF-6000, made in Japan) to measure the weight of the apple slices in regular two-minute intervals. This process was performed until reaching the final *MC* of 11 $\pm$ 1% (w.b.).

#### Moisture ratio

The moisture ratio of the apple slices was calculated accordingly to Eq. (1)^41,42^:

1 $$MR = \frac{{M_{final} - M_{eq.} }}{{M_{first} - M_{eq.} }}$$

In which, MR shows the relative moisture (dimensionless); while M_final_, M_eq._, and M_first_ denote the *MC* at time of t (d.b.), equilibrium moisture of the samples (d.b.), and initial *MC* of the slices (d.b.), respectively.

#### Specific energy consumption (SEC)

*SEC* of the drying process of the samples using the microwave method with ultrasonication and blanching pretreatment was calculated from the energy consumption of microwave method (*SEC*_1_), ultrasonication (*SEC*_2_), and blanching (*SEC*_3_). The *SEC* of the microwave drying can be obtained using Eq. (2)^5,43^:

2 $$SEC_{1} = \frac{{P_{M} t}}{{M_{w} }}$$

In which, P_M_ reveals the microwave power consumed (kW) and t represents the time of microwave use (s), While M_W_ shows the weight of the eliminated water (kg).

The *SEC* of the drying with ultrasonication pretreatment can be obtained by Eq. (3)^44^:

3 $$SEC_{2} = \frac{W.V.t}{{M_{w} }}$$

In the above equation, W shows the ultrasonic power (W/L), while V (L) and t (s), respectively, represent the volume of water and time of ultrasonication. M_W_ denotes the weight of the eliminated water (kg).

The consumption of energy of the blanching pretreatment is equal to the heat energy Q required to increase the water temperature from the ambient temperature to 80 °C which can be determined from Eq. (4). The *SEC* corresponding to this energy can be determined by Eq. (5):

4 $$Q = m.c.\Delta T$$

5 $$SEC_{3} = \frac{Q}{{M_{w} }}$$

Finally, the total energy consumption can be written as the sum of the *SEC* of ultrasonication and blanching pretreatments and microwave dryer.

6 $$SEC_{microwave + ultrasonication} \, = \,SEC_{1} \, + \,SEC_{2}$$

7 $$SEC_{microwave + blanching} \, = \,SEC_{1} \, + \,SEC_{3}$$

The energy and thermal efficiencies (TE) can be determined by Eqs. (8) and (9)^45^:

8 $$\eta_{e} = \left( {\frac{{E_{eva} }}{SEC}} \right) \times 100$$

9 $$Thermal efficincy=\frac{A.D.{h}_{fg}.({M}_{i}-{M}_{o})}{3600.t. Z. (100-{M}_{o})}$$

In the above equation, E_eva_: shows the required energy for moisture evaporation (kJ/kg), D: is the amount of product in the system (kg/m^2^), h_fg_: is the latent heat of evaporation (kJ/kg), A denotes the area of the dryer chamber (m^2^), and Z: is the heating capacity utilization (kW). In Eq. (9) for calculation of the thermal efficiency, the initial *MC* of apple (M_i_), and the mean final moisture (M_o_) of the apple were 83.2 ± $1$%, and 11$\hspace{0.17em}\pm \hspace{0.17em}1$% (w.b.), respectively.

Equation (10) shows the required energy for the evaporation of moisture from the apple and the elevation of the temperature of the samples^46^:

10 $$E_{eva} = h_{fg} .M_{w}$$

### GHG emission

Due to the production of oil and oil products—which includes the major part of exports and gross national income—Iran indirectly has a major contribution to the production of pollutants such as carbon dioxide and methane at the global level. That is because there are various types of thermal power plants (steam, gas, and combined-cycle) in Iran that cause GHG emission (NO_x_ and CO_2_). Result of other researches showed that combined cycle power plants are more efficient than other power plants^47^, thus in this research this type of power plant with different fuels (natural gas and gas oil) was investigated. GHG emission of combined cycle power plants using natural gas and gas oil for the production of 1 kWh energy was determined by coefficient of 450 and 622 g/kWh for CO_2_ and 2.95 and 3.78 g/kWh for NO_x_, respectively^48^.

### Color variation

To assess the color difference of the slices, the samples were imaged starting and finalizing the drying process by a colorimeter instrument (Portable colorimeter, model HP 200, China) to extract a*, L*, and b* parameters. These parameters represent the brightness (0–100), green to red, and blue to yellow, respectively^49^. "a" shows red-green and "b" shows yellow-blue colors. If these values are (+); "a" is red, "b" is yellow. If these values are (−); "a" is green, and "b" is blue^50^. The color variations can be determined by Eq. (11)^51–53^:

11 $$\Delta E=\sqrt{{\left({{L}_{1}}^{*}-{{L}_{2}}^{*}\right)}^{2}+{({{a}_{1}}^{*}-{{a}_{2}}^{*})}^{2}+({{b}_{1}}^{*}-{{b}_{2}}^{*}{)}^{2}}$$

In which, *a*_1_^*^, *b*_1_^*^, and *L*_1_^*^ are the color values of the dried slices with various pretreatments under various conditions. Also *a*_2_^*^, *b*_2_^*^, and *L*_2_^*^ are the color amounts of the initial samples.

### Shrinkage

The shrinkage S_*v*_ (%) was obtained by the measurement of the initial volume of the slices before drying *V*_*i*_ (cm^3^) and after drying *V* (cm^3^) by a pycnometer in the presence of toluene^38^. Then, the shrinkage was V (cm^3^) according to Eq. (12). The shrinkage can be defiend as the volume variations of the processed samples compared to the raw sampels^54^:

12 $$S_{v} = \frac{{V_{i} - V}}{{V_{i} }} \times 100$$

#### RSM

The Design Expert ver. 10 software was applied to optimize and model the drying process by a microwave dryer and ultrasonication and blanching pretreatments in terms of the thermal and quality features. To this end, RSM with “historical data” design was used to assess the effects of two independent variables of sample thickness (2, 4, and 6 mm) and microwave power (100, 200, and 300 W), and various pretreatments (control sample, 10 min of ultrasonication and blanching at 80 °C) on the dependent parameters (such as color, shrinkage, TE, DT, SEC, and EF). Independent and mutual effects on the responses were evaluated using a quadratic equation (Eq. 13)^55^:

13 $$y_{k} = \beta_{0} + \sum\limits_{j = 1}^{k} {\beta_{j} x_{j} + \sum\limits_{j = 1}^{k} {\beta_{jj} x_{j}^{2} } + \sum {\sum\limits_{i < j}^{k} {\beta_{ij} x_{i} x_{j} } } }$$

In this equation, Y shows the predicted response; while β_0_, β_i_, β_jj_, and β_ij_, respectively, denote a constant, linear coefficient, quadratic coefficient, and mutual coefficient. X_i_ and X_j_ are independent parameters. A three-dimensional response surface diagram was applied to assess the relationship between the independent and dependent parameters. Table 1 shows the coded surfaces of the independent parameters.

**Table 1:** Coded levels of independent variables and actual values corresponding to the codes.

Independent variables	Symbol	Coded levels of variables		
		− 1	− 1	+ 1
Thickness	A	2	2	6
Microwave power (W)	B	100	100	300

Among the proposed treatments of the software, the minimum DT, lowest total SEC, and lower color variation and shrinkage as well as the highest EF and TE were selected for the optimization.

## Results and discussions

### Drying time

The results of ANOVA and modeling for the assessment of the effect of the sample thickness and microwave power on the thermal and quality properties of the dried slices in a microwave dryer with different pretreatment (blanching and ultrasonication) as compared to control samples are presented in Table 2.

**Table 2:** Fitting the effect of different levels of microwave power and sample thickness on independent parameters in microwave dryer with and without pretreatment.

Pretreatment	Parameters	Equation	R^2^	Adj R^2^	Pred R^2^	CV (%)	*p* value
Control	Drying time	102.22 + 12.91A − 25.83B	0.9819	0.9759	0.9587	4.85	< 0.0001
	SEC	9.83 + 0.88 × A + 0.015 × B − 0.003AB + 0.16A^2^ − 0.0001B^2^	0.9752	0.9692	0.9581	6.71	< 0.0001
	Energy efficiency	42.40 − 2.45A − 0.03B + 0.0002B^2^	0.9570	0.9514	0.9411	4.56	< 0.0001
	Thermal efficiency	27.22–0.54A − 0.04B − 0.008AB + 0.004B^2^	0.9707	0.9654	0.9556	5.57	< 0.0001
	Color difference	16.71 + 2.25 × A + 0.03 × B − 0.003AB	0.9311	0.9221	0.9022	3.30	< 0.0001
	Shrinkage	57.71 + 1.78A − 0.04B − 0.0002B^2^	0.8975	0.8841	0.8586	3.12	< 0.0001
Ultrasound	Drying time	119.66 − 4.47A − 0.26B + 2.18A^2^	0.9572	0.9516	0.9415	7.79	< 0.0001
	SEC	10.48 + 1.87A − 0.02B − 0.003AB	0.9616	0.9566	0.9464	8.52	**< 0.0001**
	Energy efficiency	35.00 − 2.43A + 0.08B	0.9487	0.9434	0.9331	4.71	**< 0.0001**
	Thermal efficiency	29.09 − 1.51A − 0.02B − 0.004AB + 0.0003B^2^	0.9710	0.9657	0.9568	5.47	**< 0.0001**
	Color difference	13.80 + 1.45A + 0.02B	0.9310	0.9252	0.9126	3.52	**< 0.0001**
	Shrinkage	44.49 + 0.91A + 0.02B + 0.006AB	0.9179	0.9072	0.8831	3.04	**< 0.0001**
Blanching	Drying time	113.18 + 1.02A − 0.40B − 0.01AB + 1.55A^2^ + 0.0005B^2^	0.9652	0.9569	0.9420	7.46	**< 0.0001**
	SEC	8.16 + 2.03A − 0.02B − 0.0004AB	0.9604	0.9553	0.9451	9.22	**< 0.0001**
	Energy efficiency	+32.26 − 0.81A + 0.08B − 0.04A^2^	0.9568	0.9511	0.9402	4.09	**< 0.0001**
	Thermal efficiency	33.92–2.52A + 0.02B + 3.16B^2^	0.9705	0.9666	0.9594	5.25	**< 0.0001**
	Color difference	16.52 + 1.18A + 0.02B	0.8157	0.8004	0.7688	5.75	**< 0.0001**
	Shrinkage	47.85 + 1.80A + 0.04B	0.8873	0.8779	0.8573	2.82	**< 0.0001**

A: Thicknesses (mm); B: Microwave power (W); R^2^: determination coefficient; *CV*: Coefficient of variation.

Significant values are in bold.

**Figure 2:**
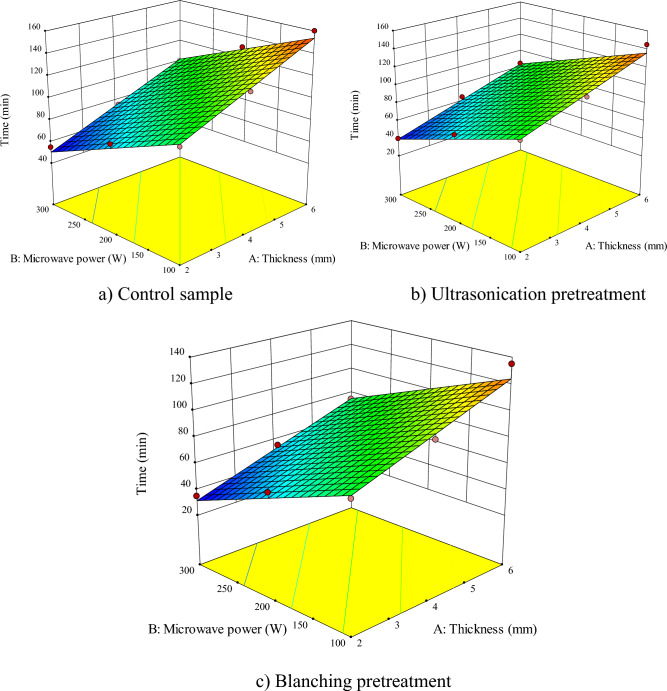
Interaction effect of sample thickness-microwave power on drying time of apple (**a**) control sample, (**b**) ultrasonication pretreatment, and (**c**) blanching pretreatment.

**Figure 3:**
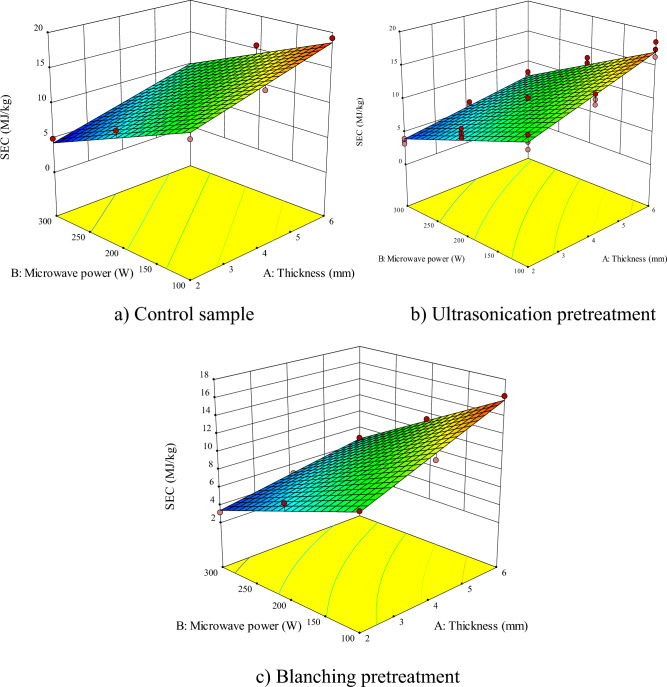
Interactive effect of sample thickness-microwave power on SEC of apple (**a**) control sample, (**b**) ultrasonication pretreatment, and (**c**) blanching pretreatment.

**Figure 4:**
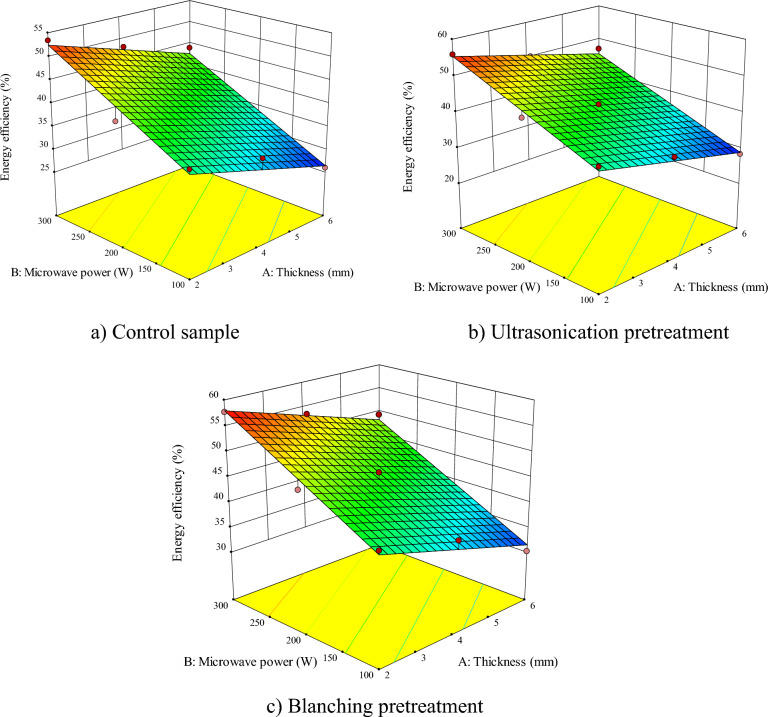
Interactive effects of sample thickness-microwave power on the energy consumption for (**a**) control sample, (**b**) ultrasonication, and (**c**) blanching pretreatments.

**Figure 5:**
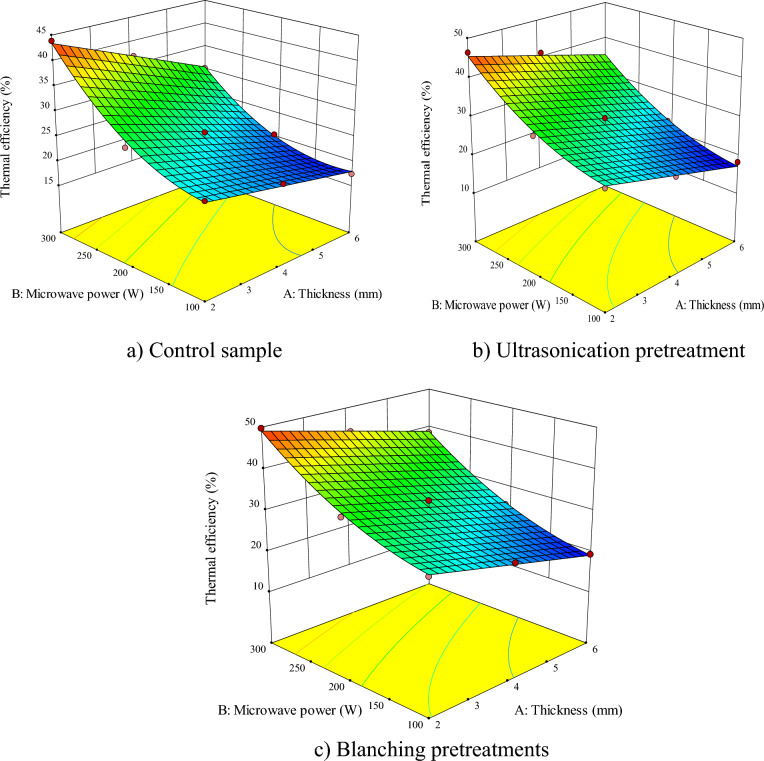
Interactive effects of sample thickness-microwave power on the thermal efficiency of dried apples (**a**) control, (**b**) ultrasonication, and (**c**) blanching pretreatments.

**Figure 6:**
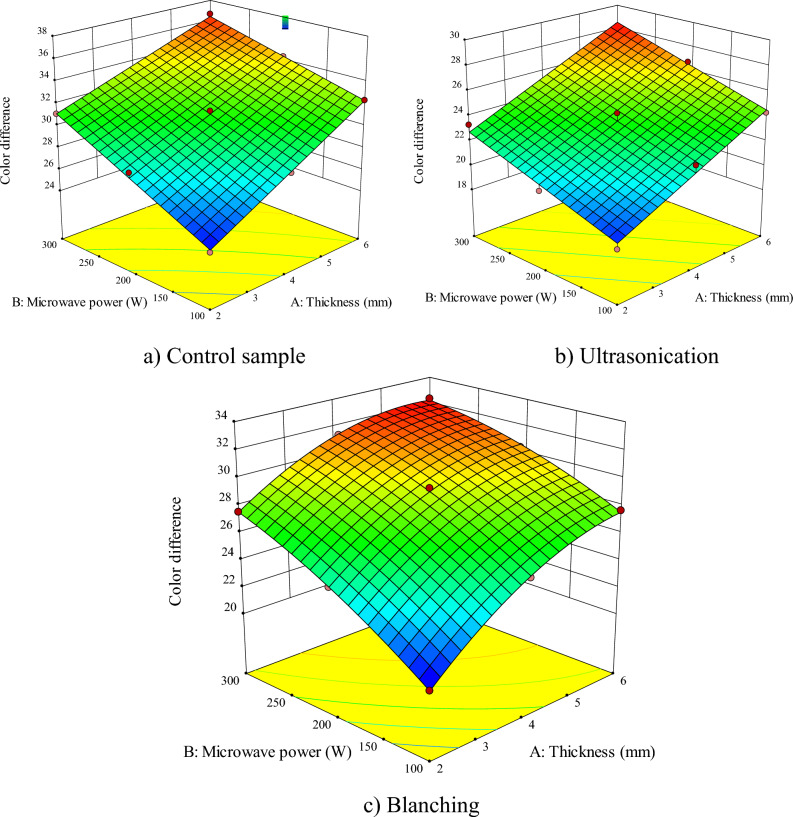
Interactive effect of sample thickness-microwave power on the color variations of apples (**a**) control, (**b**) ultrasonication, and (**c**) blanching pretreatments.

**Figure 7:**
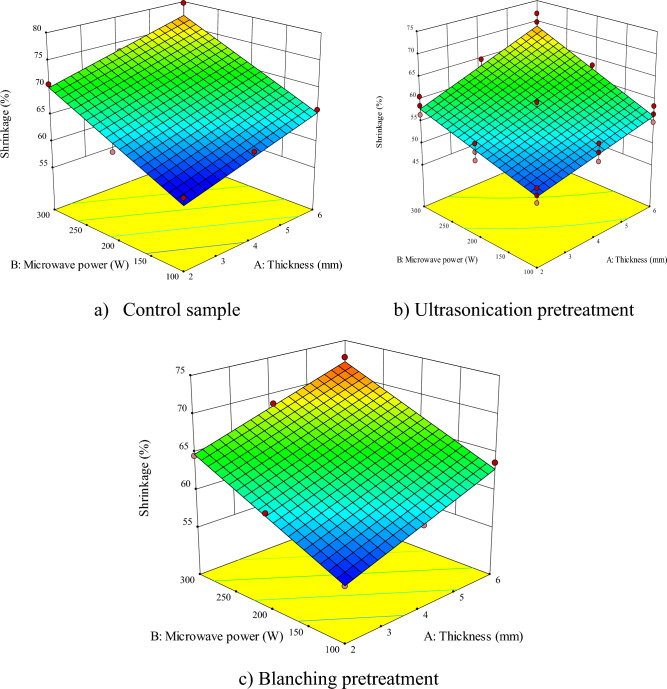
Interactive effect of sample thickness-microwave power on apple shrinkage (**a**) control, (**b**) ultrasonication, and (**c**) blanching pretreatment.

According to Figs. 2, 3, 4, 5, 6, 7 and Table 2, the proposed model is linear, quadratic, and quadratic for the untreated sample, ultrasonicated and blanched samples, respectively. As the values of Adj R^2^, R^2^, Pred R^2^ are all above 0.99, *RSM* managed to fit the best mathematical model to predict the *DT* (experimental) in the control and pretreated samples. Based on Table 2, the influences of the sample thickness and microwave power were significant for the untreated samples (*P* < 0.0001) while the mutual effects and their second order were not significant. However, for ultrasonicated samples, the effects of the independent parameters, as well as the second order sample thickness, were significant (*P* < 0.0001). Finally, for samples pretreated with blanching, the effects of all independent parameters, their mutual effects, and their second order were significant (*P* < 0.0001) on the *DT*.

Figure 2 shows the effect of sample thicknesses and microwave power on *DT* for different pretreatments. According to Fig. 2a, the shortest (55 min) and longest (160 min) *DT* were for the control sample dried at microwave power of 300 and 100 W with the thickness of 2 and 6 mm, respectively. For ultrasonic pretreatment, Fig. 2b, the longest (145 min) and shortest (40 min) drying times were for microwave power of 100 and 300 W and thicknesses of 6 and 2 mm, respectively. Based on Fig. 2c for blanching pretreatment, the minimum (35 min) and maximum (135 min) drying times were for *MP* and *ST* of 300 W—2 mm and 100 W—6 mm, respectively.

The *DT* reduced by increasing the microwave power due to faster mass transfer in the apple slices at a higher microwave power as a result of more heat generation and a large vapor pressure difference between the center and the surface of the product^56^. The *DT* also decreases by reducing the sample thicknesses as the exit of moisture will become easier, accelerating the moisture movement from the texture of the material to the sample surface^57^. Thus, drying speed increases incrementally at low thicknesses^4^. Similarly, Nagvanshi et al.^41^ and Darvishi et al.^42^ reported that microwave power and sample thickness has a significant effect on drying time.

The *DT* of ultrasonicated apple samples decreases as compared to control samples due to the formation of more microscopic channels resulting from the cavitation phenomenon^60^. The findings of this research are in agreement with the reports published by other researchers for drying potatoes^61^, carrot^24^, and Tremella fuciformis^62^ under microwave drying with ultrasound pretreatment.

Comparison of the results revealed that the use of blanching pretreatment led to a greater decrease in *DT* as compared to the use of ultrasound pretreatment and non-pretreated samples. In this case, blanched samples leads to the enzyme inactivation, oxygen removal from intercellular spaces and, therefore, a higher mass transfer^63^. Similar reports were published by other researchers for drying of different agricultural products, including white cabbage^64^, apple slices^65^, and blackberries^66^.

#### SEC

Table 2 presents the results of the ANOVA used to investigate the effect of different drying parameters (sample thicknesses and microwave power) on the *SEC* of all microwave dried apple samples with ultrasonic pretreatment and blanching. For the control samples, the effect of the *ST* and the microwave power, the interactive effect of sample thicknesses*-*microwave power, the quadratic effects of the sample thicknesses and the microwave power on the total *SEC* were significant; while for both pretreated samples, the effect of the sample thicknesses and the microwave power of the samples, the interactive effect of microwave power-sample thicknesses on the total *SEC* were significant (*P* < 0.0001).

As shown in Fig. 3, the energy consumption decreased for all three treatments by raising the microwave power and decreasing the sample thicknesses. Total *SEC* depends on different factors, such as air temperature, latent heat of water evaporation, specific heat of air, air speed, and input air temperature^67^. At higher microwave power, the free water of the product evaporates faster and the *DT* is significantly (*P* < 0.0001) declined, therefore, decreasing the total energy consumption^68,69^. The *SEC* was lower in the pretreated samples as ultrasonication leads to more destruction (creating microscopic channels) in the apple tissue, preventing the formation of the hard layer during the drying process of the pretreated samples, thus, the product dries faster and the energy consumption is reduced^70^. Other researchers reported similar results for drying layers of Aloe vera^32^, potato^61^ and onion^71^. They showed that the application of various pretreatments will facilitate the exit of moisture and reduce the *SEC*.

The reason for the increase in the *SEC* with the increase in the sample thicknesses can be assigned to the longer times required by the thicker layers to transfer their moisture from the inside to their surface for the final evaporation. This can enhance the *DT* and, in turn, the *SEC*^72^. The results of this research are compatible with the reports of other researchers for sweet potato^73,74^, kiwi^59^, and onion^5^.

According to Fig. 3, the application of blanching pretreatment decreased the *SEC* as compared to the ultrasonication because the blanching pretreatment had a direct effect on the drying speed, as it caused several cracks in the outer layer of the apple through a temperature shock^66^. As a result, the drying speed increases, therefore, less energy is required to remove moisture from the apple. Abbaspour-Gilandeh et al.^75^ used hot air and infrared methods and different pretreatments for drying terebinth; they showed that the use of blanching pretreatment requires lower *SEC* compared to the ultrasound pretreatment. Another research on drying blackberries by various dryers (infrared, hot air, and infrared—hot air) and different pretreatments concluded that the *SEC* will further reduce by using of blanching pretreatment in all three dryers as compared to ultrasound pretreatment^66^.

### Energy efficiency

Based on Table 2, the independent effects of the sample thicknesses and the microwave power as well as the second order of the microwave power are significant for the samples without pretreatment. Independent effects of the sample thicknesses and microwave power are significant for ultrasonic pretreatment; while independent effects of the sample thicknesses and the microwave power are significant for the ultrasonic pretreatment; while the independent effects of the sample thicknesses and the microwave power, as well as the second order of the sample thicknesses had significant influence on *EF* in the blanching pretreatment (*P* < 0.0001). The interactive effect of the sample thicknesses—microwave power was not significant on the *EF* in any of the pretreatments.

Figure 4a–c, respectively, show the interactive effect of sample thicknesses—microwave power on the *EF* of apple drying for control samples, ultrasound pretreatment, and blanching. The highest *EF* (57.69%) was obtained with a microwave power of 300 W and thickness of 2 mm with a blanching pretreatment, respectively. The lowest *EF* (25.69%) was for the microwave power of 100 W and thickness of 6 mm in the controlled sample, respectively. The *EF* increases by enhancing the microwave power and decreasing the sample thicknesses as they raise the value of moisture removal from the product and accelerate the evaporation of free water, hence, decreasing the *DT*. Total *SEC* also decreases due to the reduction in *DT*^69^. Maftoonazad et al.^71^ represented that using microwave/hot air hybrid drying of onion slices, the overall energy efficiency changes from about 4% to 79%. Darvishi et al.^68^ obtained the energy efficiency from 33.70 to 0.66% for soybean seeds in a microwave dryer under different powers.

The use of blanching and ultrasonication pretreatments will increase the changes in the texture of the apple and prevent the formation of the hard layer during the process of drying; therefore, the sample will be dried quicker, which will decrease the *SEC* and increase the *EF*^60^.

Namjoo et al.^76^ studied convective drying with ultrasonication pretreatment of cumin seeds and found that the EF varied from 0.689 and 1.542% for 30 ºC without pretreatment and 40 ºC with us pretreatment, respectively. Abbaspour-Gilandeh et al.^75^ showed that the maximum EF for infrared and convective drying of terebinth was obtained using ultrasonication pretreatments and blanching. Motevali et al.^77^ showed that the energy efficiency for drying apples increases by using ultrasonication and blanching.

### Thermal efficiency

Table 2 shows the significance of the independent effects of sample thicknesses and microwave power, as well as the interactive effects of the sample thicknesses*-*microwave power and second order of the microwave power on the *TE* of the untreated and ultrasonicated samples. Furthermore, the independent effect of the sample thicknesses and microwave power and second order of microwave power had a significant (*P* < 0.0001) effect on the *TE* of the blanched sample. Additionally, R^2^ values were 0.9707, 0.9710, and 0.9705 for the untreated, ultrasonicated, and blanched slices, respectively, showing the suitability of the models for predicting the *TE*.

Figure 5 depicts the interactive effects of sample thicknesses-microwave power on the *TE* for microwave drying with various pretreatments. Consequently, the highest *TE* (49.68%) was for the microwave power of 300 W and sample thicknesses of 2 mm in the blanched samples; while the lowest *TE* was obtained in the microwave power of 100 W and sample thicknesses of 6 mm, respectively, in the control samples. A rise in the microwave power and the application of pretreatments increased the *TE* as they accelerate the evaporation of the free water and enhance the mass transfer (due to the increase in the internal pressure and concentration gradient), therefore, the *DT* will be significantly shorter^77^. The *TE* showed a decline by enhancing the sample thicknesses. Since the *TE* is the ratio of the latent heat to the required energy for moisture evaporation, the rise in the product thickness is caused to decline the moisture evaporation rate, such that the input energy cannot effectively overcome the energy barrier of the sample for internal distribution of water, therefore, the *TE* will decrease. Other researchers also reported an enhancement in the energy and dryer efficiencies for thinner okra^72^ and kiwi^60^.

### Color

Table 2 lists the AVOVA results of color variation. The linear effect of the sample thicknesses and microwave power was significant on the color variations (∆E) of the apple pretreated with ultrasonication and blanching (*P* < 0.0001). The independent effects (sample thicknesses and microwave power) and the interactive effects microwave power-sample thicknesses were also significant on the color variation of the untreated apple (*P* < 0.00001).

Figure 6a–c show the effect of the sample thicknesses and microwave power on the color variation of apples with different pretreatments. The lowest and highest color variations were 18.24 and 36.67, respectively. The lowest changes were obtained for a microwave power of 100 W in ultrasonicated slices with a thickness of 2 mm. As shown in Fig. 6, by increasing the microwave power, the product is exposed to intense heat until it reaches a safe *MC* level. Therefore, a rise in the microwave power led to further increase in the color variations (∆E) between dry and fresh apples, such that a higher microwave power darkened the apples. The reason could be the formation of brown pigment through the Maillard reaction. Moreover, the increase in microwave power promoted enzymatic and non-enzymatic browning reactions, burning, and surface blackening of the samples, leading to higher color variations^65^. Other researchers reported similar results in drying garlic^12^ and mushrooms^9^ by microwave dryer and stated an incremental increase in color variations by raising the microwave power. Also, the use of pretreatment affected the color variations. The use of ultrasounds reduced the color variations as the deformation and destruction of cells and reduction of enzyme oxidation reaction occurred with the increase of ultrasound waves. These results are consistent with reports from other researchers published for kiwi^78^ and terebinth^75^. As shown in Fig. 6, a rise in the sample thickness enhanced the color variations as in thicker slices, moisture must travel a longer distance to reach the surface of the sample. This will increase the color variations of the samples^75^.

### Shrinkage

Table 2 presents the ANOVA results for shrinkage in different pretreatments. Consequently, the effect of the *ST* and the microwave power was significant (*P* < 0.0001) on the shrinkage of the samples. While the interaction effect of sample thicknesses—microwave power and the second order of microwave power were significant (*P* < 0.0001) on the shrinkage of the control and ultrasonicated samples, respectively.

Figure 7 shows the effect of the sample thicknesses and microwave power on the shrinkage of untreated, ultrasonicated, and blanched samples. The shrinkage ranged from 53.59% (for microwave power of 100 W and thickness of 2 mm, respectively, in ultrasonic pretreatment) to 73.69% (at microwave power of 300 W and thickness of 6 mm, respectively, in untreated samples).

According to the results, the simultaneous increase of the sample thicknesses and the microwave power enhanced the shrinkage. The higher shrinkage at high microwave power can be assigned to the shortening of the drying process and the application of more intense heat and humidity stresses. The shrinkage starts from the product surface and gradually reaches the inner layers by continuing the drying process^68^. Other researchers reported similar results for apple^70^ and chrysanthemum^78^.

Drying the thicker products involves the evaporation of more liquid phase (water), leading to more empty spaces inside the product, which causes a greater volume change. The other possible reason for the increase in shrinkage with the increase of the thickness can be the formation of a more porous structure with weaker bonds during the drying process due to thermal and humidity stresses which will cause more volume changes in the products^60^.

According to Fig. 7, the lowest shrinkage was related to the ultrasonicated sample. As water removal from the cell increases the stress from the liquid on the cell wall during the drying process, as a result, it will lead to the shrinkage of the material. The ultrasound pretreatment, however, will reduce the stress on the apple tissue, hence, decreasing the shrinkage^24^.

### GHG emission

**Figure 8:**
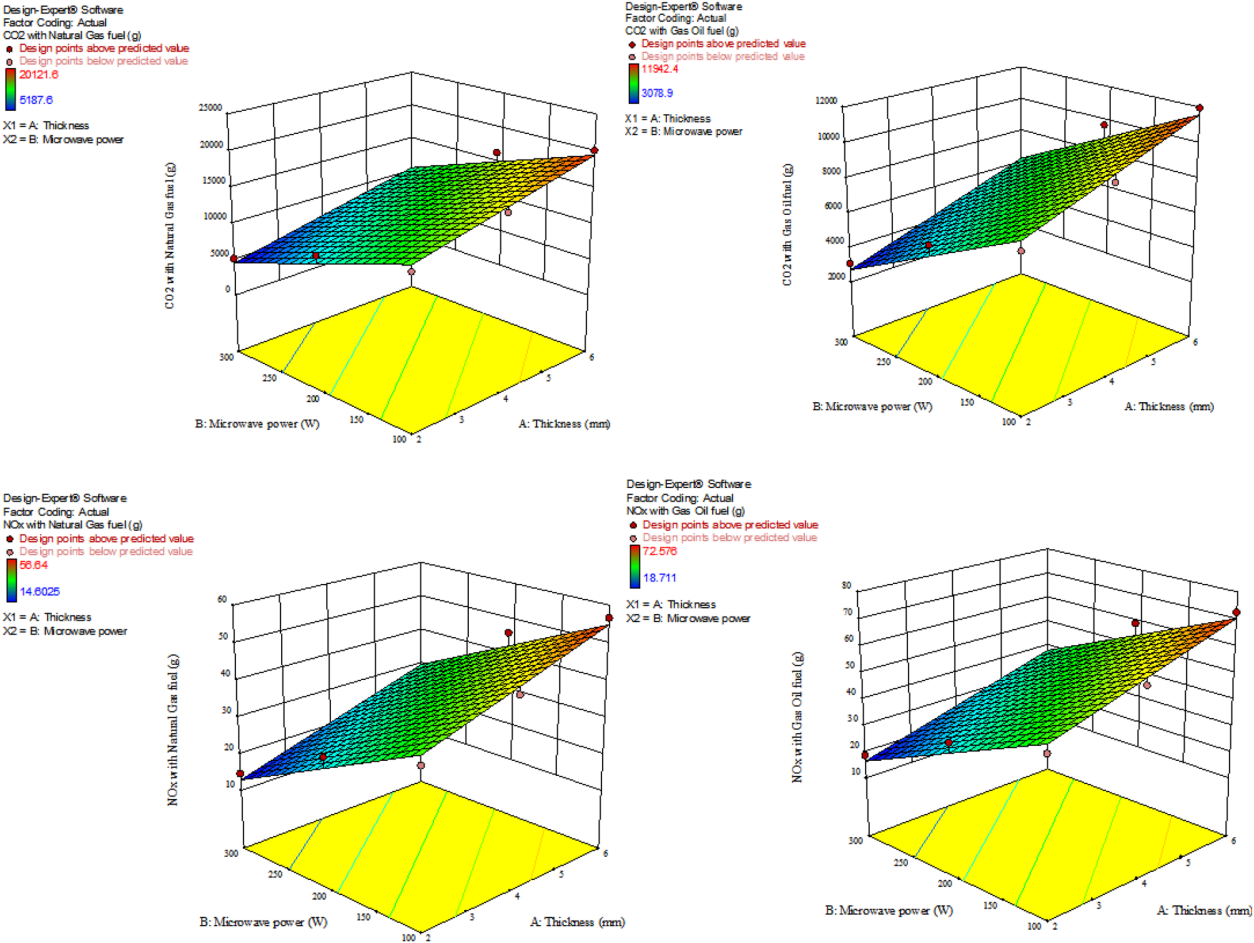
The effect of microwave power and thickness on amount of CO_2_ and NO_x_ emissions during apple drying for control samples.

**Figure 9:**
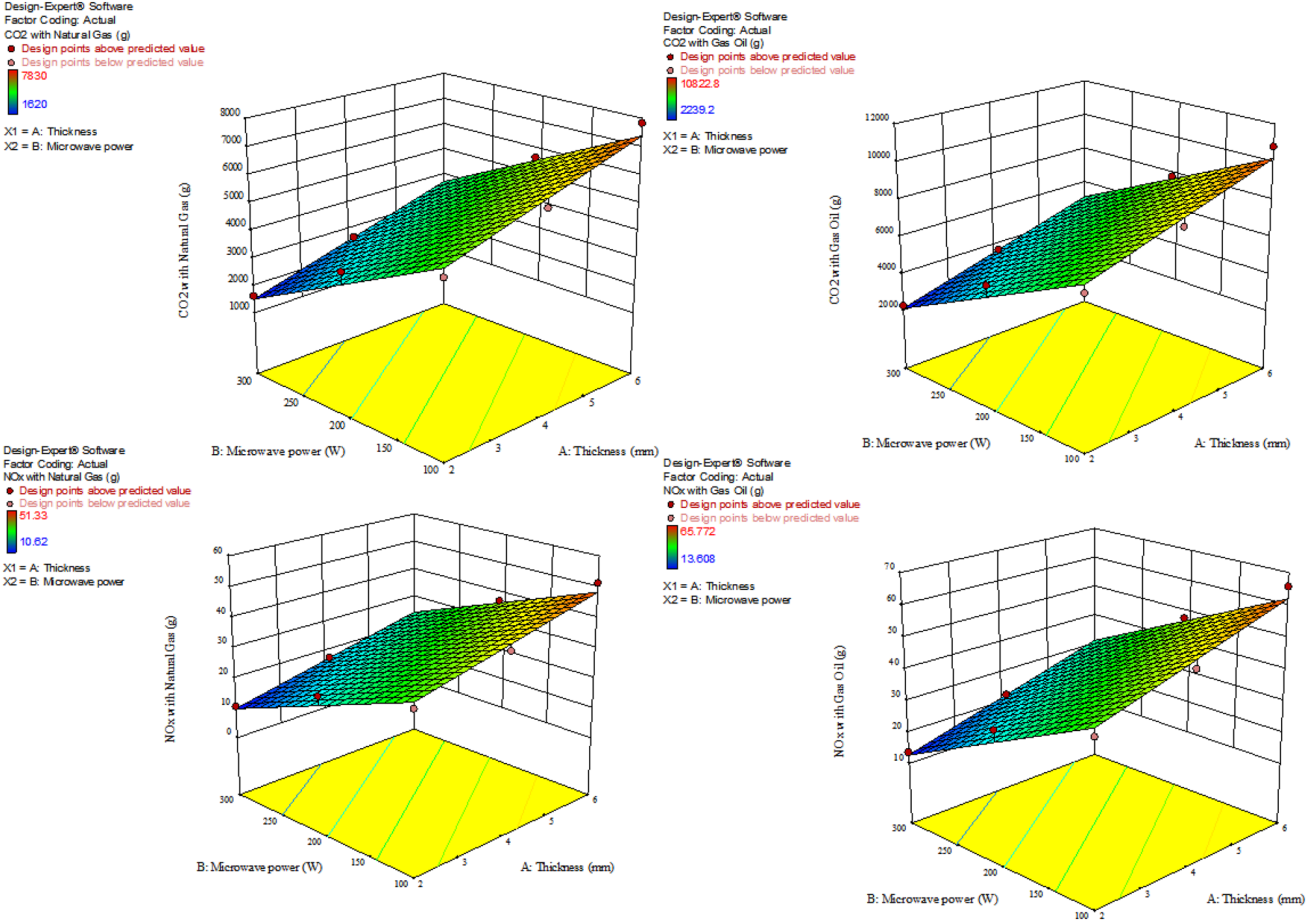
The effect of microwave power and thickness on amount of CO_2_ and NO_x_ emissions during apple drying for ultrasonication pretreatment.

**Figure 10:**
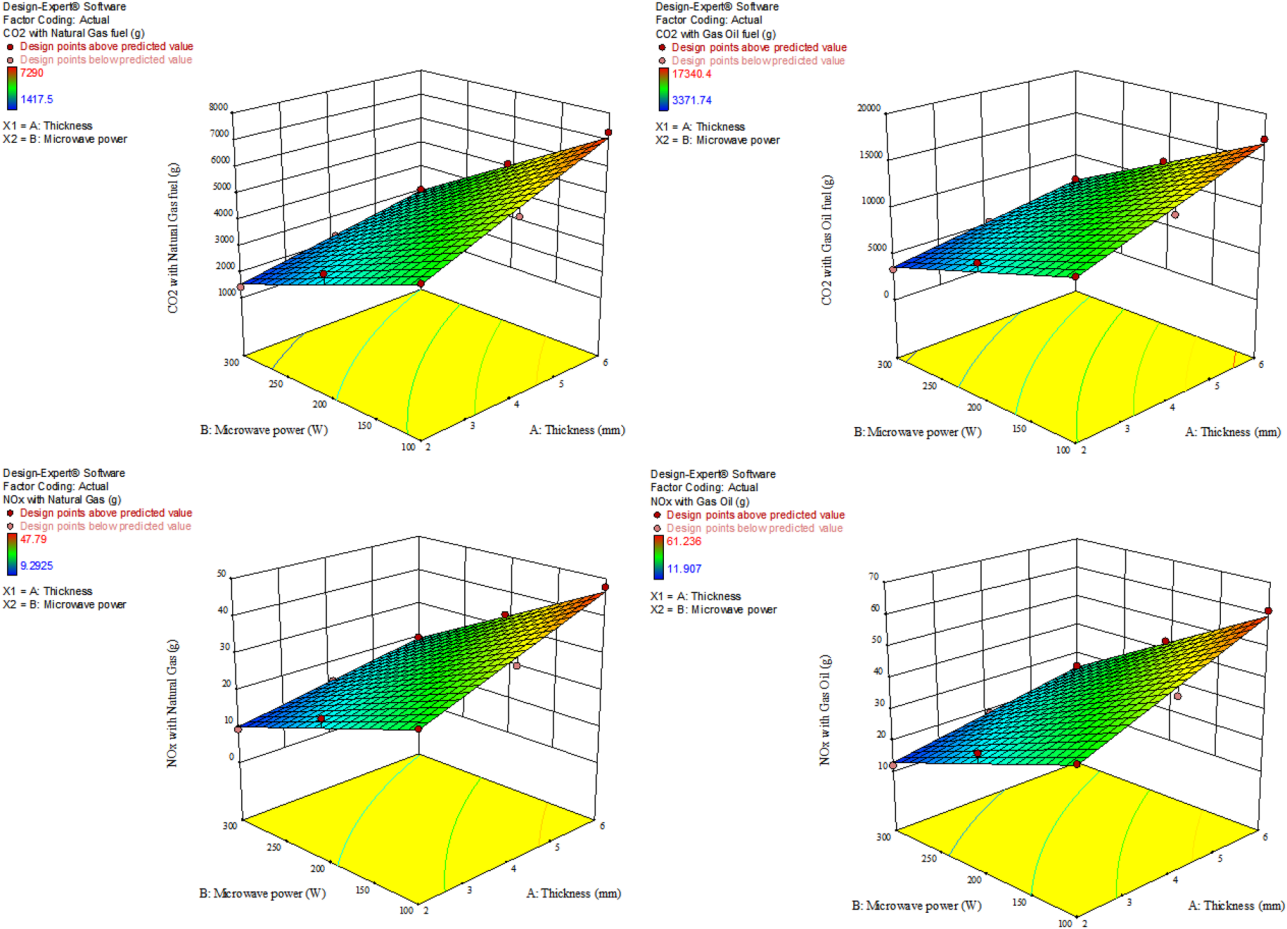
The effect of microwave power and thickness on amount of CO_2_ and NO_x_ emissions during apple drying for blanching samples.

The results of GHG modeling (CO_2_ and NO_x_) for a combined cycle power plant, as well as different pretreatments using electricity with various fuels are presented in Table 3. The R^2^ values were above 0.96. Also, the influence of the independent parameters (microwave power and sample thickness) was linearly significant (*P* < 0.0001) on the GHG emission (CO_2_ and NO_x_) for all treatments. Figures 8, 9, and 10 represent the interaction influences of microwave power and sample thickness on the CO_2_ and NO_x_ emissions based on the response surface diagrams for control, ultrasonication, and blanched samples, respectively. The highest GHG emission (CO_2_ = 20,121.6 and NO_x_ = 72.576 g) was observed in the control samples with thickness of 6 mm at the microwave power of 100 W. However, the lowest CO_2_ (1417.5 g) and NO_x_ (9.2925 g) were obtained for the drying of the blanched samples with the thickness of 2 mm at the microwave power of 300 W with natural gas fuel. According to Figs. 8, 9, and 10, it can be seen that the more the microwave power increases in fixed thicknesses, the more GHG emission decreases. Motevali and Koloor^80^, Motevali et al.^32^ and Kaveh et al.^81^ stated that the process of reducing GHG emission with increasing microwave power was due to the reduction in energy consumption and drying time of the samples. While the increase in thickness in constant powers caused an increase in GHG emission. Seyfi et al.^33^ showed that increasing the thickness of Aloe vera gel increases GHG emission for different drying methods due to the increase in drying time. Taghinezhad et al.^24^ and Kaveh et al.^81^ also obtained similar results for drying carrots and pear in different thicknesses, respectively.

**Table 3:** Fitting the effect of different levels of microwave power and sample thickness on CO_2_ and NO_x_ values in microwave dryer.

Pretreatment	Type of fuel	Equation for CO_2_	R^2^	Equation for NOx	R^2^
Control	Natural gas	14,934 + 1506.5A − 44.54B	0.9636	42.038 + 4.24063A − 0.12538B	0.9636
	Gas oil	8863.5 + 894.125A − 26.435B	0.9636	53.865 + 5.43375A − 0.16065B	0.9636
Ultrasound	Natural gas	5872.5 + 556.875A − 18.225B	0.9743	38.4975 + 3.65063A − 0.11948B	0.9743
	Gas oil	8117.1 + 769.725A − 25.191B	0.9743	49.329 + 4.67775A − 0.15309B	0.9743
Blanching	Natural gas	3675 + 913.5A − 9.56250B − 1.8225A.B	0.9858	24.09167 + 5.9885A − 0.06288B − 0.011947 A.B	0.9858
	Gas oil	8741.55 + 2172.89849A − 22.74586B − 4.33509A.B	0.9858	30.87 + 7.6734A − 0.080325B − 0.015309A.B	0.9858

A: Thicknesses (mm); B: Microwave power (W); R^2^: determination coefficient; *CV*: Coefficient of variation.

### Optimization

*RSM* is one of the most important mathematical methods for optimizing agricultural dried samples as it straightly affects the selection of the best drying parameters^82^. The optimization results for samples dried in a microwave dryer under different pretreatments are shown in Table 4 with desirability. The response variables included color, shrinkage, total SEC, EF, DT, TE, CO_2_, and NO_x_. Accordingly, optimal drying conditions involve the microwave power of 300 W and *ST* of 2 mm for all treatments, respectively. The optimization findings show the positive effect of microwave power on the response variables as they grew with the enhancement of microwave power. Therefore, the optimal microwave power was considered to be 300 W. The thickness was also suggested at its minimum level to decrease the *DT* and due to its positive effect on the response variables.

**Table 4:** Results of optimization by desirable function of RSM with the best thickness of 2 mm and microwave power of 300 W.

Pretreatment	Time (min)	SEC (MJ/kg)	Energy efficiency (%)	Thermal efficiency (%)	Color difference	Shrinkage (%)	Desirability
Control	50.55	4.37	52.44	43.41	31.13	70.11	0.921
Ultrasonication	39.44	3.37	55.40	45.50	22.73	59.42	0.935
Blanching	31.55	3.42	57.97	49.07	27.56	64.81	0.916

## Conclusions

This research addressed the optimization of the thermal properties and quality of apples dried in a microwave dryer under ultrasonication and blanching pretreatments with the help of *RSM*. Independent parameters included sample thickness and microwave power while response variables included *SEC,* drying time, energy efficiency, thermal efficiency, color, shrinkage, GHG emission. The results revealed a significant effect of independent parameters on all response variables in all pretreatments (*P* < 0.0001). The optimal values of independent variables involved microwave power of 300 W and sample thickness of 2 mm, respectively. Additionally, the desirability index was 0.921, 0.935, and 0.916 for untreated, ultrasonicated, and blanched samples, respectively. Analysis of variance was carried out to check the accuracy of the model fit. The variation analysis and surface graphs showed the effect of factors on the responses where the increase of microwave power and the use of different pretreatments had a positive effect on reducing *SEC, GHG* emissions, and drying time while increasing the energy and thermal efficiency of the drying. Moreover, the use of thinner apple slices was especially useful for reducing the shrinkage and color changes. Today, the development of post-harvest quality inspection approaches, especially, with the aim of optimization and using novel techniques to reduce processing time as well as *GHG* emissions can decrease the wastes and achieve standardization.

## Data Availability

All data generated or analyzed during this study are included in the article. All authors read and approved the final manuscript.

## List of symbols

*A*: Area of the dryer chamber (m^2^)
*C*_*m*_: Specific heat of the samples (kJ/kg K)
*D*: Sample’s density of loading (kg/m^2^)
*DT*: Drying time (min)
*E*_*eva*_: The required energy for moisture evaporation (kJ)
*EF*: Energy efficiency (%)
*E*_*heating*_: Energy required for elevating the temperature of the product (kJ)
*h*_*fg*_: Latent heat of evaporation (kJ/kg)
*MC*: Moisture content (%)
*M*_*eq.*_: Equilibrium moisture of the samples (d.b.)
*M*_*final*_: Moisture content at time of t (d.b.)
*M*_*first*_: Initial moisture content of the samples (d.b.)
*M*_*i*_: Initial *mc* of apple
*M*_*o*_: Final moisture of the apple
*MP*: Microwave power (W)
*M*_*W*_: Weight of eliminated water (kg)
*M*_*W*_: Amount of the evaporated moisture (kg)
*P*_*M*_: Microwave power consumed (kW)
*RSM*: Response surface methodology
*SEC*: Specific energy consumption (kJ/kg)
*ST*: Sample thickness (mm)
*S*_*v*_: Shrinkage (%)
*t*: Time (s)
*T*_*1*_: Temperature of the samples before drying (K)
*T*_*2*_: Temperature of the samples during drying (K)
*TE*: Thermal efficiency (%)
*V*: Water volume (L)
*Vi*: Sample volume before and after drying (cm^3^)
*W*: Ultrasonic power (kJ/L)
*W*_*d*_: Weight of the dry matter (kg)
*Y*: Response variable
*Z*: Heat transfer rate (kW)
*ε*: Error
$$\Delta E$$: Colour variation
